# Hansen’s disease masquerading as rheumatoid arthritis: A case report

**DOI:** 10.1002/ccr3.5822

**Published:** 2022-05-12

**Authors:** Prashant Pant, Oshan Shrestha, Pragyat Singh, Vivek Paudyal, Istiyaque Alam, Prabhat Singh Rajput, Dhan Bahadur Shrestha

**Affiliations:** ^1^ Department of Anesthesia and Critical Care Karnali Academy of Health Sciences Jumla Nepal; ^2^ 475258 Nepalese Army Institute of Health Sciences Kathmandu Nepal; ^3^ Department of Otorhinolaryngology Karnali Academy of Health Sciences Jumla Nepal; ^4^ Department of Dermatology Karnali Academy of Health Sciences Jumla Nepal; ^5^ Department of Internal Medicine Mount Sinai Hospital Chicago Illinois USA

**Keywords:** arthritis, Hansen's disease, leprosy, musculoskeletal manifestation

## Abstract

Hansen's disease, a chronic granulomatous disease, classically has cutaneous and neurological manifestations. Musculoskeletal manifestation of the disease is underappreciated. This case report conveys that musculoskeletal manifestation of the disease should not be missed, as sometimes it is the only presenting symptom, to avoid delay in correct diagnosis and treatment.

## Graphical abstract

Key messages of this case remains that musculoskeletal manifestations of the Hansen's disease should not be missed by clinicians.

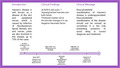

## 1 INTRODUCTION

Hansen's disease is well known in today's date as a disease of the skin and peripheral nerves, which is caused by infection of *Mycobacterium leprae (M*. *leprae)* or *Mycobacterium lepromatosis (M*. *lepromatosis)*. Although this chronic granulomatous disease classically presents with cutaneous and neural involvement, musculoskeletal involvement has also been identified but is underrecognized and underreported.^1^ The involvement of joints in leprosy may be underreported but occurs in up to 75% of the cases, and at times, it happens to be the only presenting manifestation.^2^ In this case report, we share case details of a 71‐year‐old gentleman who had rheumatoid arthritis‐like features as presenting manifestation of leprosy. This case report is in line with the CARE guidelines.^3^

## 2 CASE REPORT

A 71‐year‐old gentleman, a permanent resident of a rural village of Nepal, was referred to our tertiary level healthcare center with the concern of nonhealing ulcers over his both hands. Thorough history‐taking of the patient revealed his past medical history of being diagnosed with rheumatoid arthritis (RA) ten years back. This diagnosis of RA was based on the American College of Rheumatology/European League Against Rheumatism (ACR/EULAR) rheumatoid arthritis classification criteria.^4^ The patient had swelling and pain on multiple joints (more than 10 joints; including joints of both hands, feet, and knees), for more than six weeks, and abnormal erythrocyte sedimentation rate. Thus, obtaining a total score of 7, the diagnosis of RA was made and was prescribed methotrexate (2.5 mg, once a week) and folic acid (5 mg, once a week) in the past. The patient was lost to follow‐up to his treating doctor as his symptoms improved. After five years of diagnosis of RA, the patient started to lose sensation over his both hands (distal to wrist) and feet (distal to ankle). The patient reported losing his slippers while walking as he would not feel them. The patient also reported handling hot utensils with his bare hands as he would not feel the burning sensation, and the patient also experienced cigarette burns due to this. This led to the development of blisters and ulcers over his hands from time to time. Initially, these ulcers healed, but later on, they were not healing and recurrent infection troubled the patient. The patient was referred from the local healthcare center to our tertiary level hospital when discharge started to come from the ulcer.

The patient has no history of hypertension, diabetes mellitus, thyroid disorders, tuberculosis, stroke, nor does he have a history of any surgical intervention in the past. However, he has a smoking history of 15 pack years. There is no significant family history that could indicate his condition.

### 2.1 Timeline

**FIGURE. 1:**
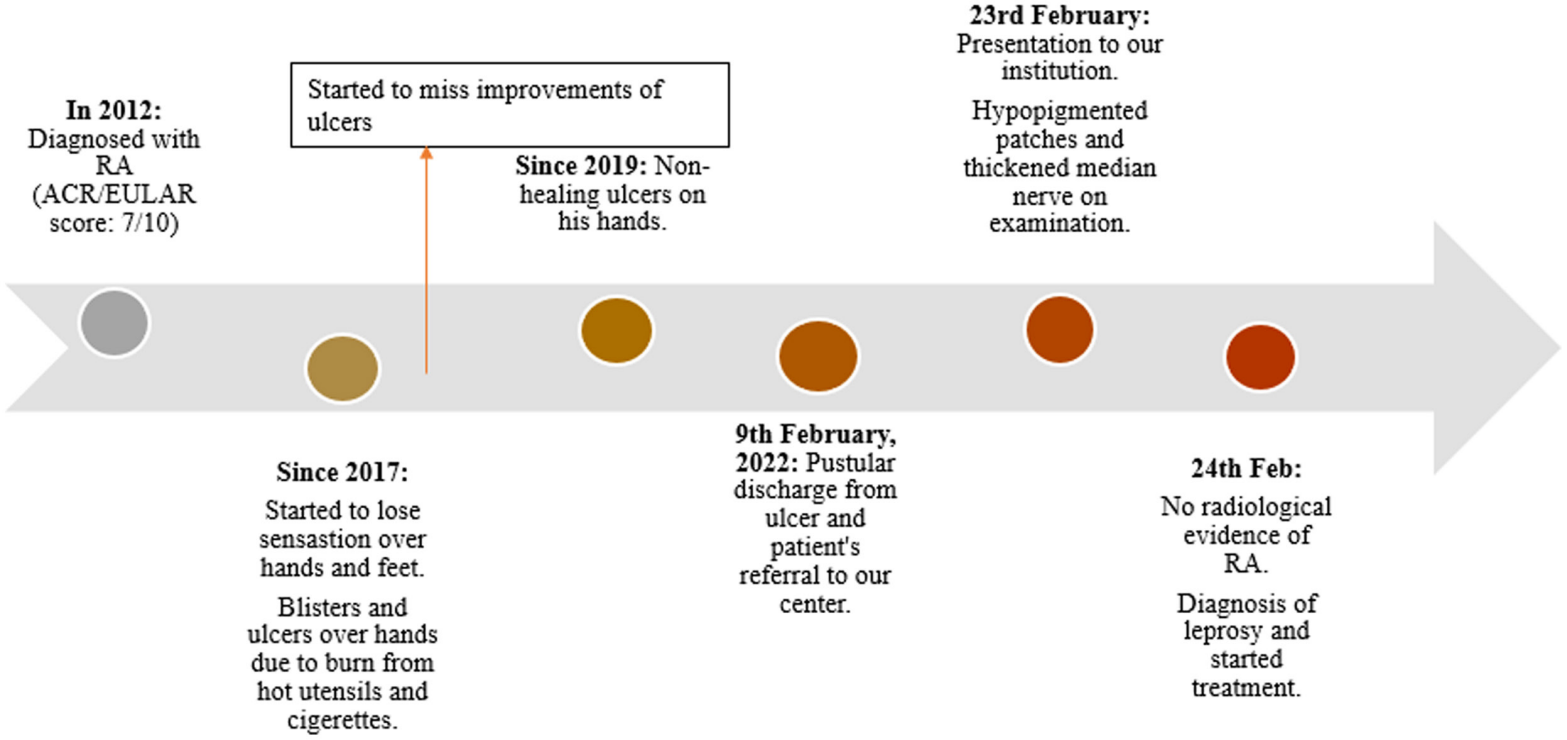
Timeline showing major events

Clinical evolution of the patient spans in the duration of ten years. The major clinical events that happened over these ten years are summarized in the timeline (Figure 1).

### 2.2 Diagnostic assessments

**FIGURE. 2:**
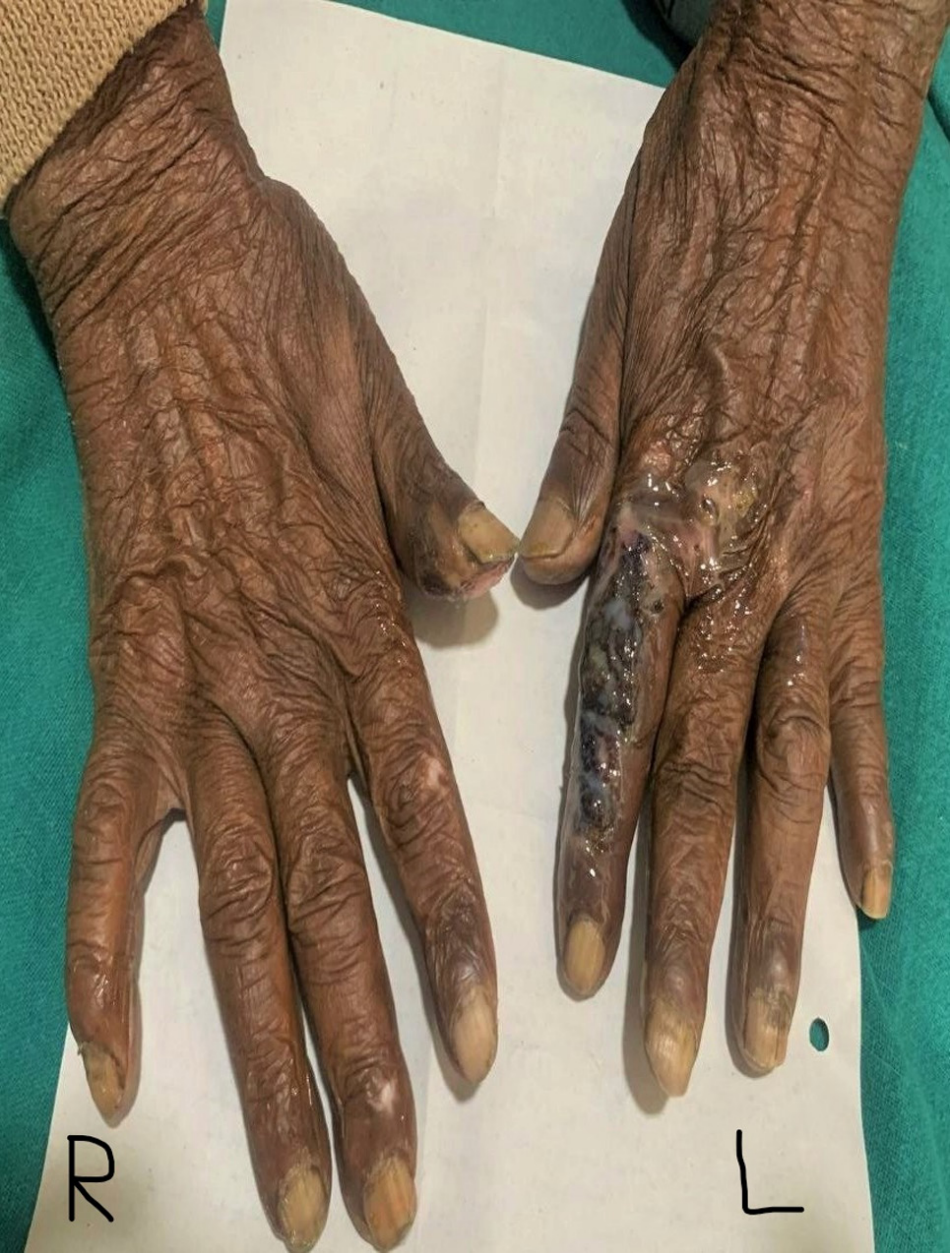
Linear ulcer of size 7 cm × 2 cm over the dorsal aspect of the left index finger

**FIGURE. 3:**
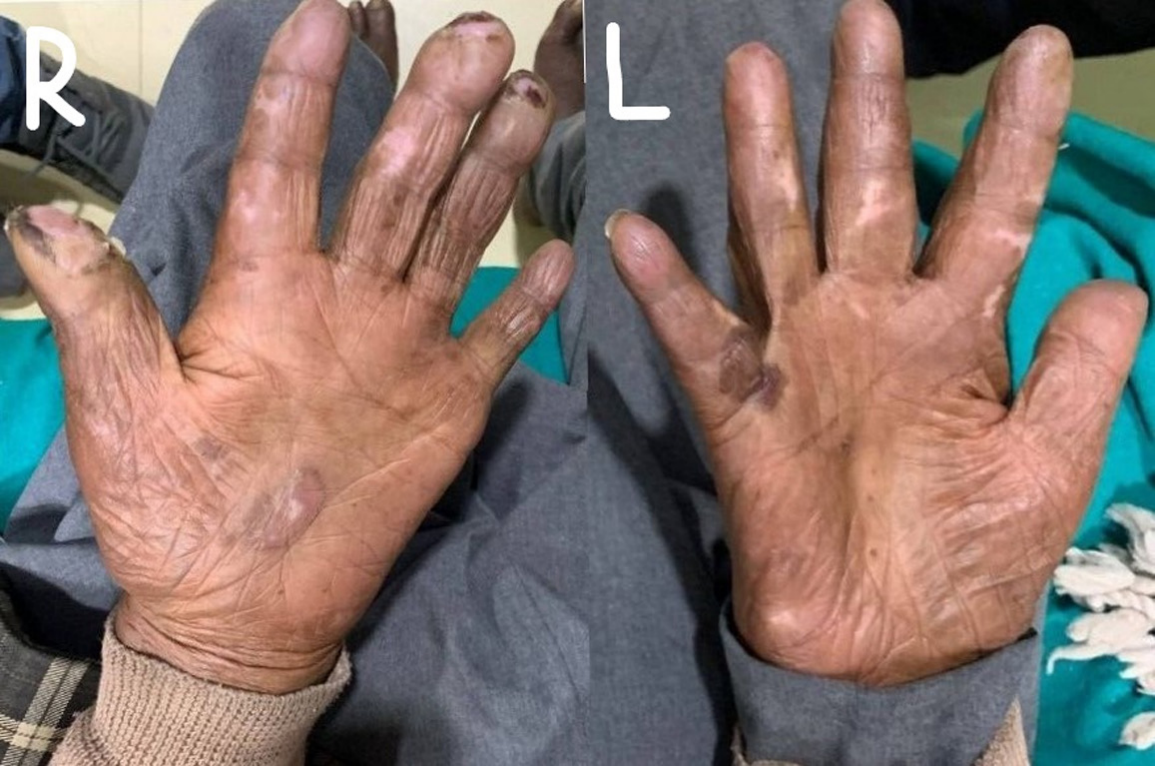
Multiple hypopigmented lesions over right and left hands

Physical examination of the patient, on the day of presentation, showed an oval punched‐out ulcer, non‐tender on palpation, with a diameter of 2 centimeters (cm) located over the pulp of the right thumb with no granulation tissue or discharge was found. Similar ulcers over pulp of right middle and right ring fingers of diameters of 1.5 cm and 1 cm, respectively, and a blister of size 1 cm over the proximal phalanx of the right little finger were noted. On examining the left hand, a linear ulcer of size 7 cm × 2 cm over the dorsal aspect of the left index finger, which was firm and non‐tender on palpation (Figure 2), with overlying crusts, no discharge, was found. Peeling of skin over palm on the right hand was also noted. The patient also had multiple hypopigmented macules (more than 5 in number) in both hands (Figure 3).

**FIGURE. 4:**
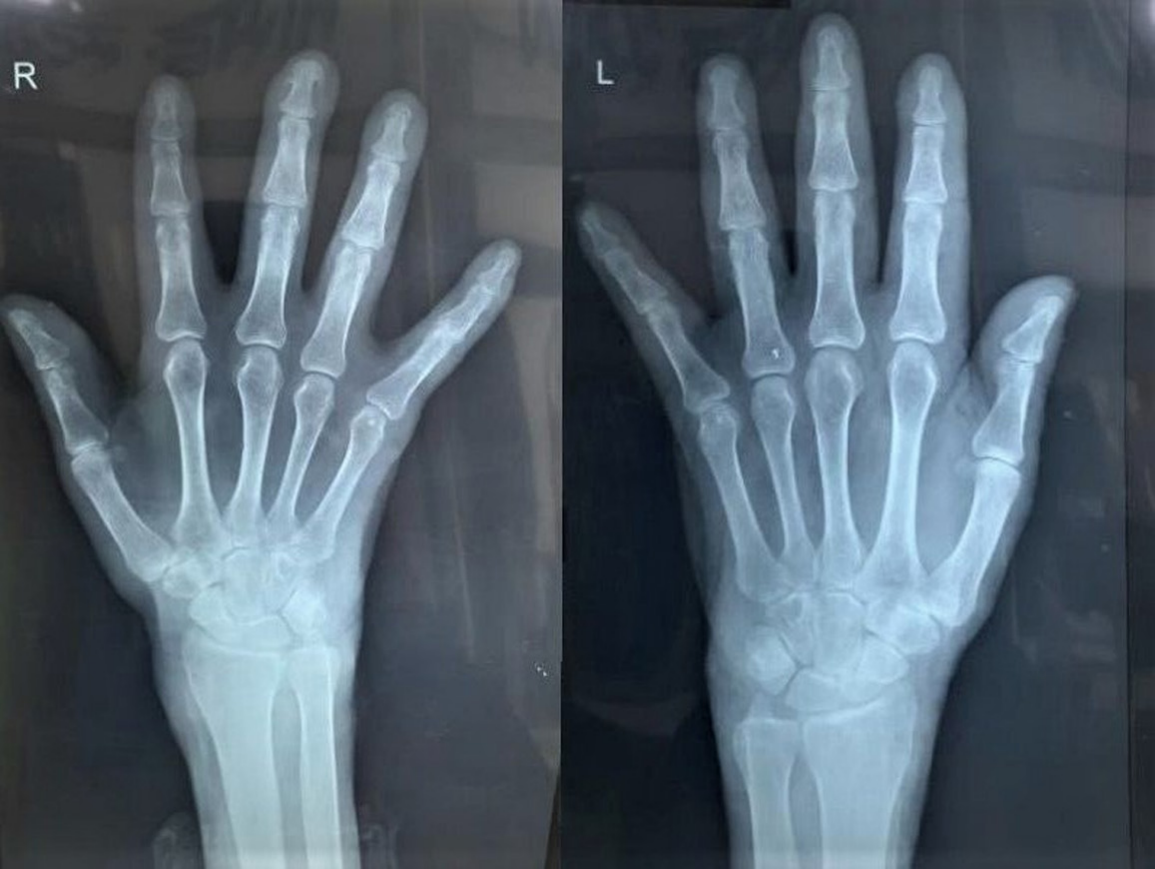
Radiological findings noncompliant with rheumatoid arthritis

Neurological examination revealed complete loss of touch, pain, and temperature sensation over bilateral hands and feet with intact motor function. On palpation, the median nerve on the left forearm was thickened. However, other neurological assessments had normal findings, and the power of hand was normal. While examining the oral cavity, an ulcer with underlying granulation tissues at the base of the uvula on the right side which was tender on palpation was found. Radiological investigations of the hands showed no articular changes that could point to rheumatoid arthritis. (Figure 4) Laboratory findings of the patient on the day of presentation are shown in Table 1.

**TABLE 1:** Laboratory findings

Complete Blood Count	
Hemoglobin	12.8 g/dl
Red blood cell count	4.4 millions/ml
Total leucocyte count	8600/cm^3^
Differential count	(N: 80%, L: 13%, E: 5%, M: 2%)
Platelets	184,000/cm^3^
Biochemistry	
Random blood sugar	82.4 mg/dl
Total bilirubin	0.8 mg/dl
Alkaline phosphatase	550 µ/L
AST	68.2 µ/L
ALT	39.5 µ/L
Urea	25.3 mg/dl
Creatinine	1.1 mg/dl
Sodium	139.8 mEq/L
Potassium	4.3 mEq/L
Urine Routine examination	
Albumin	Nil
Glucose	Nil
Pus cells	2–4/high power field
Red blood cells	Nil
Epithelial cells	3–4/ high power field
Casts	Nil
Hematology	
C‐reactive protein	Positive
Erythrocyte sedimentation rate	40 mm/h
Serology	
Rheumatoid factor	Negative

Abbreviations: cm, centimeter; dl, deciliter; E, eosinophils; g, gram; L, leucocytes; l, liter; M, monocytes; mEq, milliequivalent; mg, milligram; ml, milliliter; mm, millimeter; N, neutrophils; u, units.

With the consideration of current practice in diagnosing leprosy, which is based on the presence of at least one of the three cardinal features (definite loss of sensation in a hypopigmented patch; thickened or enlarged peripheral nerve; the presence of acid‐fast bacilli in a slit‐skin smear),^5^ the absence of articular changes in the X‐ray, and negative rheumatoid factor, clinical diagnosis of Hansen's disease with trophic ulcer was made.

### 2.3 Treatment

The patient was admitted to the dermatology ward and was started on anti‐leprotic drugs (multibacillary—multidrug therapy) for one year (dapsone, clofazimine, and rifampicin), anti‐bacterial drugs (cefadroxil), multivitamin capsule (thiamine, riboflavin, pyridoxine, nicotinamide, calcium pantothenate, cyanocobalamin, folic acid, ascorbic acid, and zinc sulfate), and topical emollient (coconut oil). Daily wound dressing was performed for his ulcers. Analgesic (paracetamol and ibuprofen combined tablet), chlorhexidine gargle, and ointment (combination of lignocaine, metronidazole, and chlorhexidine) were also given to the patient for the oral cavity ulcer.

### 2.4 Follow‐up

The patient is currently under treatment. Despite the advice of staying at the hospital, the patient requested discharge. The patient is counseled about his condition, and he is told about the importance of follow‐up. The patient is called for a follow‐up after two weeks.

## 3 DISCUSSION

This case report presents a case of delayed diagnosis of Hansen's disease due to unusual presentation. In this patient, cutaneous manifestation was preceded by musculoskeletal manifestation. Articular symptoms in Hansen's disease show great variability among studies; however, it is seen as the third commonest after dermatological and neurologic manifestation.^1^, ^6^ There exists a similarity between patients of Hansen's disease and rheumatoid arthritis as joint manifestation can be seen and is sometimes the only presenting manifestation.^2^ In such cases, recognition of rheumatic manifestation in Hansen's disease becomes crucial. Dermatological and neurological manifestations of Hansen's disease are well known, but musculoskeletal manifestations are underappreciated. Dermatological manifestations include macules, papules, plaques, or nodules, which may be hypopigmented and anesthetic and the neurological manifestations include mononeuropathy, mononeuritis multiplex, or peripheral neuropathy.^2^ Neurological manifestation can be thickened nerves and a spectrum of neuropathies.^7^ While musculoskeletal manifestation includes arthritis. One study has reported arthritis in 54.28% of leprosy patients and has also reported that leprosy can present with arthritis as the first symptom.^8^ However, there is no formal classification for the musculoskeletal manifestations despite being the third most common symptoms of Hansen's disease. Henriques et al have suggested a classification for arthritis in leprosy; acute arthritis of lepra reaction and chronic arthritis.^9^ Acute arthritis occurs as a part of lepra reactions, whereas chronic arthritis is an entity with rheumatoid arthritis‐like distribution and features.^10^, ^11^, ^12^

ACR/EULAR RA classification criteria is a classification system that focuses on features that develop at earlier stages of the disease rather than a late‐stage feature of the disease.^4^ Although our patient had a score of 7 of 10 according to ACR/EULAR rheumatoid arthritis classification criteria, which backs up the diagnosis of rheumatoid arthritis, the patient had no articular changes that are compliant with the disease. Joint space narrowing, fusion of joints, and dislocation are the typical manifestations of RA seen in the X‐ray. Radiographic findings of the patient showed no such features even after ten years of joint involvement history points out the importance of comprehensive assessment of clinical, laboratory, and radiological features. With this being said, it is also important to consider that RA can coexist with Hansen's disease.^13^

This case also reinforces the importance of follow‐up. The patient had lost follow‐up to his treating doctor, if not, he could have been diagnosed with Hansen's disease much earlier. Losing follow‐up is a common practice in Nepal, especially in rural parts. Stopping the follow‐up when the symptoms get better or just continuing the medicine on their own is a major problem in the country. In today's date, also Nepal is highly reliable on hand‐written documents and is not compliant with the system of storing the information in the online databases. And, when patients lose their documents, their past illnesses and past medications cannot be traced accurately. Through this case report, we would also like to remind the concerned authorities for policy revisions to mitigate such third‐world countries’ problems.

## 4 CONCLUSION

The clinical manifestations of Hansen's disease can sometimes be not so apparent. Joint involvement of the disease should not be overlooked as it can be the first presenting complaint and missing it may take the diagnosis and treatment in the line of rheumatological conditions. A comprehensive assessment of clinical findings, laboratory findings, radiological findings, and regular patient follow‐up is helpful to avoid delays in diagnosis and treatment.

## AUTHOR CONTRIBUTIONS

Prashant Pant (PP) and Oshan Shrestha (OS) contributed to the conception and design of the study. PP, Pragyat Singh (PS) Vivek Paudyal (VP), and Istiyaque Alam (IA) contributed to acquiring patients’ detailed information. OS performed the literature review and contributed to initial manuscript drafting. Prabhat Singh Rajput (PSR) and Dhan Bahadur Shrestha (DBS) guided throughout the study and edited intellectually. All authors were involved in drafting and revising the manuscript and approved the final version.

## CONFLICT OF INTERESTS

No conflicts of interests.

## ETHICAL APPROVAL

Not applicable.

## CONSENT

Written informed consent was obtained from the patient for the publication of this case report and accompanying images. A copy of the written consent is available for review by the Editor‐in‐Chief of this journal on request.

## PATIENT PERSPECTIVE

The patient is anxious about his condition but is assured that he will get better as the right treatment has been started. The patient is positive and has promised to come for the follow‐up.

## CLINICAL TRIAL REGISTRATION

Not applicable.

## Data Availability

All the findings are present within the manuscript.
